# Gender- and Age-Specific Associations Between Body Fat Composition and C-Reactive Protein with Lung Function: A Cross-Sectional Study

**DOI:** 10.1038/s41598-018-36860-9

**Published:** 2019-01-23

**Authors:** Yu-Chung Tsao, Yi-Yen Lee, Jau-Yuan Chen, Wei-Chung Yeh, Chung-Hsun Chuang, Wei Yu, Wen-Cheng Li

**Affiliations:** 1 Department of Occupation Medicine, Chang-Gung Memorial Hospital at Linkou, Taoyuan, Taiwan; 2 Department of Family Medicine, Chang-Gung Memorial Hospital at Linkou, Taoyuan, Taiwan; 3 Division of Pediatric Neurosurgery, Neurological Institute, Taipei Veterans General Hospital, Taipei, Taiwan; 4 Faculty of Medicine, National Yang-Ming University, Taipei, Taiwan; 5 Department of Emergency Medicine, Xiamen Chang-Gung Hospital, Xiamen, China; 6 Department of Health Management, Xiamen Chang-Gung Hospital, Xiamen, China; 7 Department of Emergency Medicine, Chang-Gung Memorial Hospital at Linkou, Taoyuan, Taiwan

## Abstract

Lung function deterioration is frequently observed in overweight and obese patients. In the current study, we explored the contribution of body fat (BF) composition, particularly visceral and nonvisceral adiposity, to lung function deterioration. In addition, we examined gender- and age-specific differences in the association between the joint effects of BF% and C-reactive protein (CRP) concentrations with lung function. This cross-sectional study involved 17,802 subjects undergoing health check-up. Clinical characteristics, body composition using bioelectrical impedance analysis (BIA), and lung function were evaluated and compared between genders. Subjects were stratified by gender-specific BF% cut-offs for evaluating the association of body composition and the risk of restrictive lung disease (RLD). Gender differences in the joint effects of BF% and CRP on lung function were observed. Visceral obesity increased the risk of RLD in women aged ≥45 years, but nonvisceral obesity reduced the risk of RLD in women aged <45 years. Our findings suggest that visceral fat *per se* can be used as an effective parameter to assess the association between lung function and obesity.

## Introduction

Obesity is considered a worldwide epidemic and is predicted to affect more than 1 billion people by 2020^1^. Studies have demonstrated that obesity is associated with morbidity and mortality; specifically, visceral or central obesity poses a higher risk of a clustering of disorders^2^, including cardiovascular events, diabetes, and cancers^2–5^. Impaired lung function is also frequently observed in overweight and obese subjects^6–11^.

Excess adipose tissue in the abdominal region exerts a mechanical effect on the lungs by reducing the capacity of the diaphragm to limit lung inflation, consequently diminishing chest wall movement^10^. Adipose tissue function and distribution are distinctly different between men and women^12^. Men are more prone to visceral obesity, and women have a higher percentage of body fat^13^. The regional distribution of body fat is a distinct characteristic of sexual differentiation and may account for some of the sex differences observed in lung mechanics^14^. Women generally have a higher percentage of body fat than men do.

Adipose tissues not only store triglycerides (TG) but also secrete various signalling molecules and hormones and contribute to metabolic homeostasis. Visceral fat releases different bioactive molecules, hormones, and proinflammatory cytokines, and it triggers increased expression of C-reactive protein (CRP)^15^. CRP is an acute-phase protein and serves as a marker of systemic inflammation^12^. Therefore, visceral obesity is associated with a low-grade inflammatory state^16^. Studies have reported that individuals with obesity had higher CRP serum concentrations compared with those of normal weight^17,18^. By contrast, caloric restriction weight-loss diet reduced serum CRP level in postmenopausal women^19^.

Peripheral CRP is naturally higher in women compared with men, independent of age, race, and body mass index (BMI)^20^. Furthermore, studies have indicated that higher androgen and lower oestrogen concentrations have an anti-inflammatory effect in men^21^. Previous studies have reported that CRP is associated with reduced lung function^22^ and faster rate of forced expiratory volume in 1 s (FEV_1_) decline and cardiovascular disease^23^. Considering the role of inflammation in lung function, visceral fat decreases lung function through a different mechanism in addition to the mechanical effects of visceral fat *per se*.

We investigated how deterioration in lung function is affected by the coexistence of high fat accumulation and how systemic subclinical inflammation is associated with restrictive pulmonary function. Because body fat distribution is markedly different between men and pre- and postmenopausal women, the present study explored the contribution of body fat composition and distribution, particularly visceral and nonvisceral adiposity, to lung function deterioration. Furthermore, we examined gender- and age-specific differences in the association between the joint effects of body fat percentage (BF%) and CRP on lung function.

## Methods

### Subjects

This was a retrospective cross-sectional study of adults aged ≥18 years who attended annual health examinations at three medical centres of Chang-Gung Memorial Hospital during 2014–2015. The study was approved by the Institutional Review Board of Chang-Gung Memorial Hospital and was conducted in accordance with the guidelines laid down in the Declaration of Helsinki. As a retrospective chart review, we reviewed existing data from the patients’ charts. The information recorded could not identify the subjects. No information enabling the identification of any subject and no linking list of any sort were recorded. Therefore, informed consent for study participation was not applicable in the current study.

Participants were surveyed using questionnaires to assess their medical history (disease and medication histories) and physiological conditions (including pregnancy and fasting time) during health examination. Trained nurses were responsible for collecting data and venous blood. All information was coded electronically on a central record database. Participants with missing data or who met the following criteria were excluded from analysis: (1) without a minimum of 12-h fasting prior to blood sampling; (2) pregnant; (3) diagnosed with chronic diseases that could significantly affect pulmonary function, such as chronic obstructive pulmonary disease, myocardial infarction, angina pectoris, asthma, tuberculosis, and lung cancer; (4) with a serum hsCRP level >10 mg/L that indicated the presence of an inflammatory or infectious state.

### Measurements

Body weight and height were measured to the nearest 0.1 kg and 0.1 cm. BMI was calculated as weight divided by height squared (kg/m^2^). Blood pressure was measured thrice using an automated sphygmomanometer after the participant was in the seated position for at least 15 min. Up to three measurements were averaged for systolic and diastolic pressures. Mean arterial pressure (MAP) was typically estimated using an approximation equation: 2/3*diastolic pressure + 1/3* systolic pressure. Clinical chemistry workup included fasting plasma glucose (FPG), total cholesterol (TC), TG, LDL cholesterol (LDL-C), and HDL cholesterol (HDL-C). The rate turbidity method was used to measure serum CRP concentrations through high sensitivity CRPH reagent assays on a Beckman DxC 800 Automatic Analyzer (Beckman Coulter, Inc., Fullerton, CA, USA). Blood tests were conducted in accordance with the laboratory SOP of the hospital, which was accredited by the College of American Pathologists.

BF% was measured using a BIA (MC 180 model; Tanita, Tokyo, Japan). For accurate measurement of BF%, all subjects were instructed not to exercise or consume alcohol for at least 24 h prior to examining.

### Lung function tests

Spirometry was used to measure lung function, which was performed by specially trained technicians according to the 1994 American Thoracic Society recommendations^24^ using a dry rolling seal spirometer (Spirolab III; Medical International Research, Rome, Italy). We analysed only data from subjects with three or more acceptable spirometry performances. Values measured in the study were forced vital capacity (FVC), FEV_1_, and FEV_1_ to FVC ratio (FEV_1_/FVC) in accordance with the definition by the American Thoracic Society. Restrictive lung disease (RLD) was defined as FVC < 80% and FEV_1_/FVC > 0.7 of predicted value.

### Statistical analysis

Demographic and clinical characteristics for continuous variables were presented as mean and standard deviation, and those for categorical variables were expressed as number and percentage. The 75th percentile values for the cut-off point of BF% and CRP were used to assess the combined effects of inflammation and body fat accumulation on restrictive pulmonary function. Four groups were categorised as follows: (1) reference group (BF% < 75th percentile and CRP < 75th percentile); (2) high CRP group (BF% < 75th percentile and CRP ≥ 75th percentile); (3) high BF% group (BF% ≥ 75th percentile and CRP < 75 percentile); and (4) high CRP and high BF% group (BF% ≥ 75th percentile and CRP ≥ 75th percentile). Analyses were conducted separately for men and women aged <45 years and ≥45 years.

The mean value of continuous clinical characteristics between the genders was compared using an independent sample *t* test. The trend of clinical characteristics across the four study groups (the reference, high CRP, high BF%, and high CRP/BF% groups) was tested using linear contrast in the general linear model for continuous parameters or using a Cochran–Armitage chi-squared test for categorical parameters (i.e. prevalence of RLD).

The subjects were stratified by gender-specific BF% cut-offs according to the definitions of the Chinese Taipei Association for the study of obesity (http://www.ctaso.org.tw/index0.htm) as follows: (1) low BF%, men BF% < 17% and women BF% < 20%; (2) normal BF%, men BF% 17–23% and women BF% 20–27%; and (3) high BF%, men BF% > 23% and women BF% >27%. To investigate the joint effects of body fat distribution pattern on RLD, we created a variable of three groups categorised according to BF% and visceral fat degree as follows: (1) nonobese type, BF% ≤ 27; (2) visceral obesity type, BF% > 27 and visceral fat degree >10; and (3) nonvisceral obesity type, BF% > 27 and visceral fat degree ≤10. The categorised groups were then used as independent variables of interest in the multivariable logistic regression models in which potential confounding factors, including waist-to-height ratio (WHtR), MAP, FPG, and hsCRP levels, were sequentially adjusted for. Data analysis was performed using SPSS 22 (IBM SPSS, Armonk, NY: IBM Corp).

## Results

A total of 17,802 subjects were included in this study. Mean ages were similar between genders (Table 1). Men were characterised by a more deteriorated metabolic risk profile than women were because they had significantly higher levels of the measured clinical parameters, including BMI, WHtR, MAP, FPG, TC, TG, LDL-C, and TG/HDL-C ratio, whereas the result of HDL-C was reversed (*P* < 0.001). Women had a significantly higher BF% than men did (21.5% vs. 30.1%, *P* < 0.001). The measurements of hsCRP indicated low-grade inflammation for both genders, though the inflammation level was significantly higher for male than that for female (2.10 vs. 1.44 μg/mL, *P* < 0.001). The functional parameters of the respiratory system showed that men had significantly lower values of volume parameters (FVC% and FEV_1_%) and FVC%/FEV_1_% ratio than women did (*P* < 0.001).

**Table 1:** Main characteristics of the study subjects by gender (n = 17,802).

Characteristics	Men	Women	*P* value
Number (%)	9,652 (54.2)	8,150 (45.8)	—
Age, years	42.8 (10.8)	42.8 (11.3)	0.895
BMI (kg/m^2^)	24.5 (3.3)	22.4 (3.3)	<0.001
Waist-to-height ratio	0.51 (0.05)	0.49 (0.06)	<0.001
Body fat percentage, %	21.5 (5.5)	30.1 (6.1)	<0.001
Mean arterial pressure (mmHg)	91.2 (12.2)	83.5 (12.1)	<0.001
Fasting glucose (mmol/L)	5.35 (1.39)	5.12 (0.94)	<0.001
Total cholesterol (mmol/L)	5.22 (0.97)	4.99 (0.96)	<0.001
Triglycerides (mmol/L)	1.76 (1.56)	1.08 (0.95)	<0.001
LDL cholesterol (mmol/L)	3.38 (0.85)	3.04 (0.81)	<0.001
HDL cholesterol (mmol/L)	1.22 (0.27)	1.47 (0.31)	<0.001
TG/HDL-C	1.63 (2.03)	0.83 (1.13)	<0.001
hsCRP (μg/mL)	2.10 (5.14)	1.44 (3.17)	<0.001
FVC, % predicted	89.8 (12.0)	92.7 (13.4)	<0.001
FEV_1_, % predicted	93.2 (12.0)	95.0 (13.4)	<0.001
FEV_1_/FVC ratio	86.2 (6.3)	87.9 (6.1)	<0.001

Data are presented as mean (SD) or percentage (SD).

BMI, body mass index; TG, triglycerides; HDL-C, HDL cholesterol; hsCRP, high sensitivity. C-reactive protein; FVC, forced vital capacity; FEV_1_, forced expiratory volume in 1 second.

**Figure 1:**
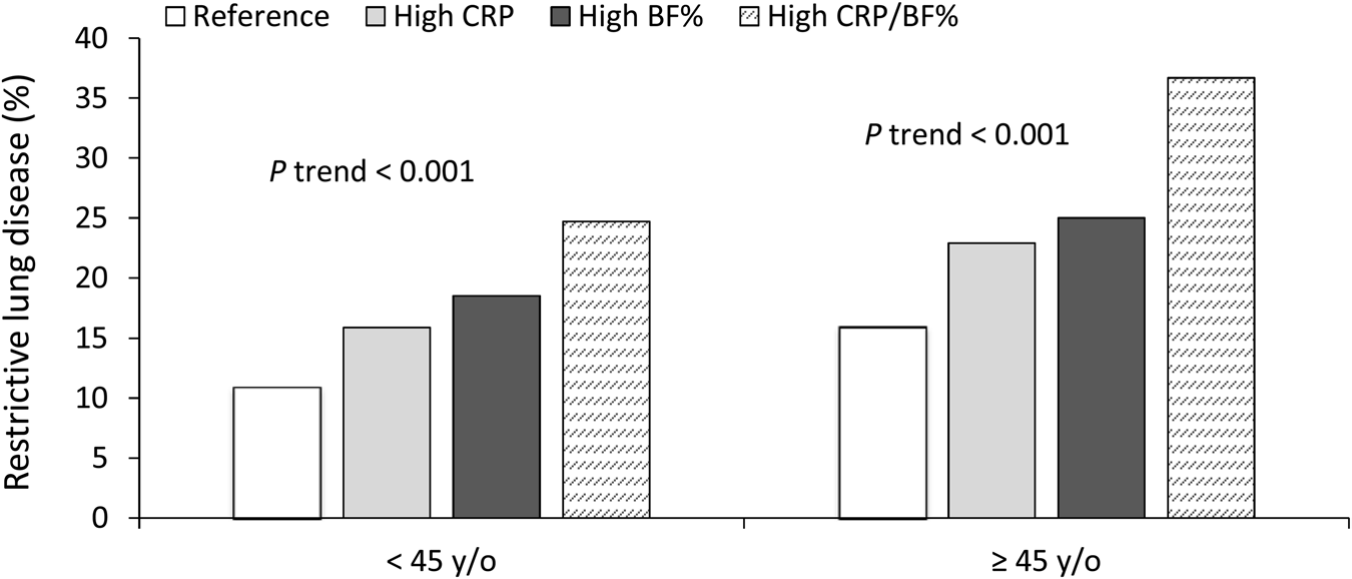
Prevalence of restrictive lung disease according to CRP and BF% stratified by age (<45 and ≥45 years) in men. The groups were categorised into (1) reference group (BF% < 75th percentile, CRP < 75th percentile); (2) high CRP group (BF% < 75th percentile, CRP ≥ 75th percentile); (3) high BF% group (BF% ≥ 75th percentile, CRP < 75th percentile); and (4) high CRP and high BF% group (BF% ≥ 75th percentile, CRP ≥ 75th percentile).

In men aged <45 years (Table 2), significant differences were observed in all clinical measurements between the reference group, high CRP group (inflammatory group), high BF% group (obese group), and high CRP and high BF% group (inflammatory and obese group); this change exhibited an increasing trend toward the group with high CRP and high BF%, except for age and FVC%/FEV_1_% ratio. No significant trend across the four groups was detected for FVC%/FEV_1_% ratio in men of all ages. An increasing trend of RLD prevalence was also observed across both the age groups. A similar observation was noted in groups aged ≥45 years, but the overall prevalence was higher in the older age groups than it was in the younger age groups (Fig. 1).

**Table 2:** Baseline characteristics of the study subjects according to CRP and body fat percentage stratified by age in men.

Characteristics	CRP < 75% fat% < 75%	CRP ≥ 75% fat% < 75%	CRP < 75% fat% ≥ 75%	CRP ≥ 75% fat% ≥ 75%	*P* value (ANOVA)	*P* value (P trend)
**<45** **y/o**						
Number	3624	834	842	643	—	—
Age, years	35.7 (5.3)	35.7 (5.1)	36.3 (5.4)	35.9 (5.3)	0.060	0.175
BMI (kg/m^2^)	23.0 (2.6)	23.9 (2.7)	28.0 (2.5)	29.1 (3.0)	<0.001	<0.001
Waist-to-height ratio	0.48 (0.04)	0.50 (0.04)	0.55 (0.04)	0.57 (0.04)	<0.001	<0.001
Body fat percentage, %	18.8 (4.4)	20.3 (3.9)	27.7 (3.3)	28.8 (3.1)	<0.001	<0.001
Mean arterial pressure (mmHg)	88.4 (10.7)	90.3 (11.5)	95.4 (11.5)	98.3 (12.5)	<0.001	<0.001
Fasting glucose (mmol/L)	5.04 (0.87)	5.33 (1.65)	5.34 (1.26)	5.62 (1.65)	<0.001	<0.001
Total cholesterol (mmol/L)	5.09 (0.92)	5.16 (1.00)	5.38 (0.98)	5.43 (1.00)	<0.001	<0.001
Triglycerides (mmol/L)	1.52 (1.26)	1.94 (2.20)	2.19 (1.67)	2.51 (2.22)	<0.001	<0.001
LDL cholesterol (mmol/L)	3.27 (0.80)	3.35 (0.87)	3.53 (0.81)	3.58 (0.93)	<0.001	<0.001
HDL cholesterol (mmol/L)	1.28 (0.28)	1.16 (0.24)	1.13 (0.21)	1.09 (0.19)	<0.001	<0.001
TG/HDL-C	1.33 (1.67)	1.91 (3.07)	2.07 (1.98)	2.47 (2.66)	<0.001	<0.001
hsCRP (μg/mL)	0.71 (0.48)	5.90 (9.79)	1.03 (0.49)	5.45 (7.16)	<0.001	<0.001
FVC, % predicted	91.8 (11.5)	89.4 (11.6)	88.9 (11.2)	86.7 (11.4)	<0.001	<0.001
FEV_1_, % predicted	94.8 (11.3)	92.0 (11.4)	91.7 (10.9)	89.5 (11.2)	<0.001	<0.001
FEV_1_/FVC ratio	87.0 (6.2)	86.8 (5.9)	86.8 (5.7)	86.8 (5.6)	0.658	0.452
Restrictive lung disease, n (%)^†^	395 (10.9)	133 (15.9)	156 (18.5)	159 (24.7)	<0.001	<0.001
**≥45** **y/o**						
Number	2212	590	572	335	—	—
Age, years	53.5 (6.9)	55.1 (7.7)	54.7 (8.5)	54.7 (8.3)	<0.001	0.022
BMI (kg/m^2^)	23.4 (2.4)	23.8 (2.4)	27.4 (2.3)	28.0 (2.6)	<0.001	<0.001
Waist-to-height ratio	0.50 (0.04)	0.51 (0.04)	0.56 (0.04)	0.58 (0.04)	<0.001	<0.001
Body fat percentage, %	19.4 (4.1)	20.4 (4.0)	27.6 (2.2)	28.5 (3.1)	<0.001	<0.001
Mean arterial pressure (mmHg)	89.7 (12.2)	91.9 (13.1)	97.2 (12.8)	98.8 (14.1)	<0.001	<0.001
Fasting glucose (mmol/L)	5.45 (1.44)	5.77 (1.70)	5.81 (1.72)	6.15 (2.26)	<0.001	<0.001
Total cholesterol (mmol/L)	5.25 (0.93)	5.39 (1.09)	5.30 (0.99)	5.46 (1.03)	<0.001	0.003
Triglycerides (mmol/L)	1.57 (1.18)	1.87 (1.99)	1.99 (1.39)	2.23 (1.66)	<0.001	<0.001
LDL cholesterol (mmol/L)	3.39 (0.82)	3.52 (0.93)	3.44 (0.87)	3.58 (0.94)	<0.001	0.002
HDL cholesterol (mmol/L)	1.26 (0.29)	1.16 (0.27)	1.14 (0.23)	1.10 (0.22)	<0.001	<0.001
TG/HDL-C	1.39 (1.38)	1.88 (3.07)	1.89 (1.59)	2.21 (2.12)	<0.001	<0.001
hsCRP (μg/mL)	0.80 (0.52)	7.10 (11.46)	1.05 (0.53)	5.45 (6.29)	<0.001	<0.001
FVC, %predicted	90.3 (12.1)	87.8 (13.1)	86.3 (11.9)	82.6 (12.2)	<0.001	<0.001
FEV_1_, %predicted	94.6 (12.6)	91.9 (13.8)	91.1 (12.6)	86.9 (13.1)	<0.001	<0.001
FEV_1_/FVC	85.1 (6.6)	84.6 (6.3)	85.4 (6.3)	85.0 (6.1)	0.226	0.758
Restrictive lung disease, n (%)^†^	355 (16.0)	135 (22.9)	143 (25.0)	123 (36.7)	<0.001	<0.001

^†^Chi-squared test or Cochran–Armitage test for trend.

BMI, body mass index; TG, triglycerides; HDL-C, HDL cholesterol; FVC, forced vital capacity; FEV_1_, forced expiratory volume in 1 s. CRP, C-reactive protein.

Data are presented as mean (SD).

**Figure 2:**
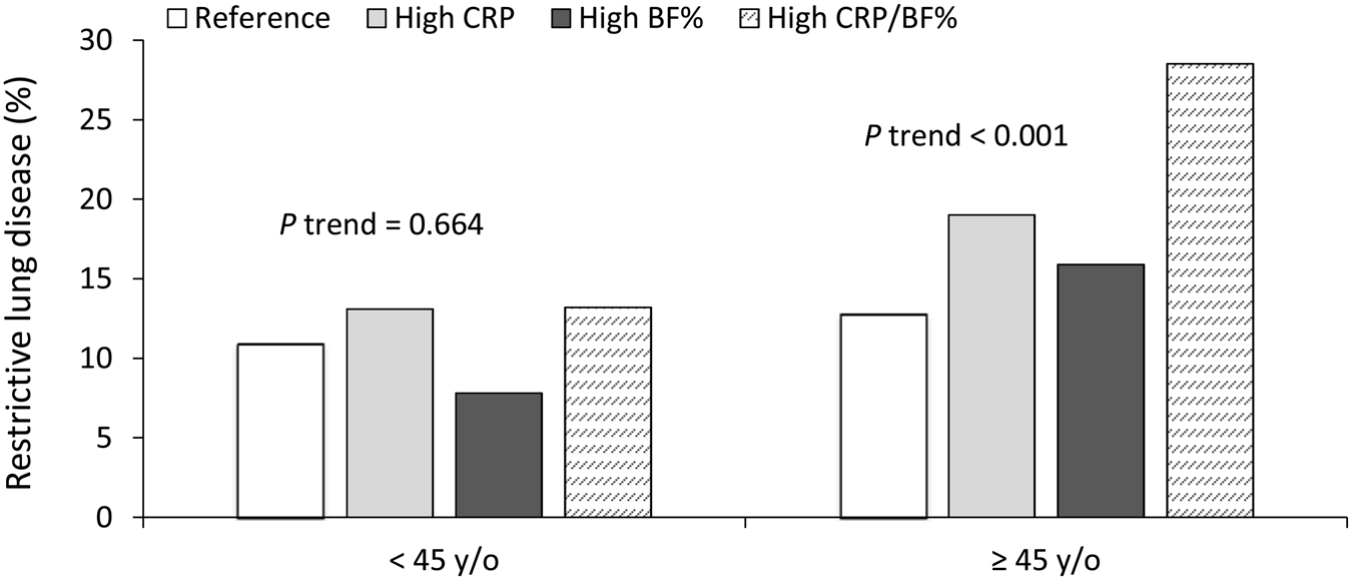
Prevalence of restrictive lung disease according to CRP and BF% stratified by age (<45 and ≥45 years) in women. The groups were categorised into (1) reference group (BF% < 75th percentile, CRP < 75th percentile); (2) high CRP group (BF% < 75th percentile, CRP ≥ 75th percentile); (3) high BF% group (BF% ≥ 75th percentile, CRP < 75th percentile); and (4) high CRP and high BF% group (BF% ≥ 75th percentile, CRP ≥ 75th percentile).

In the analysis for women aged <45 years (Table 3), trend changes were generally similar in clinical measurements as observed in men. However, change in trend of FVC% across the younger age groups and that of FVC%/FEV_1_% ratio across the older age groups was not observed. The prevalence of RLD was generally lower in women than it was in men (Table 2). Women aged ≥45 years had higher CRP levels than those aged <45 years. The trend change patterns of RLD prevalence, waving in ways consistent with the changes of CRP, were different from those of men of comparative groups (Fig. 2).

**Table 3:** Baseline characteristics of the study subjects according to CRP and body fat percentage stratified by age in women.

Characteristics	CRP < 75% fat% < 75%	CRP ≥ 75% fat% < 75%	CRP < 75% fat% ≥ 75%	CRP ≥ 75% fat% ≥ 75%	*P* value (ANOVA)	*P* value (P trend)
**<45** **y/o**						
Number	3034	641	600	570	—	—
Age, years	34.4 (5.3)	34.8 (5.4)	36.4 (5.1)	36.3 (5.2)	<0.001	<0.001
BMI (kg/m^2^)	20.1 (1.9)	20.8 (1.9)	24.4 (2.1)	25.7 (2.9)	<0.001	<0.001
Waist-to-height ratio	0.44 (0.04)	0.46 (0.04)	0.50 (0.04)	0.53 (0.05)	<0.001	<0.001
Body fat percentage, %	25.9 (4.1)	27.4 (3.6)	34.9 (2.5)	36.6 (3.8)	<0.001	<0.001
Mean arterial pressure (mmHg)	78.9 (9.1)	79.6 (9.2)	81.8 (10.2)	85.0 (12.1)	<0.001	<0.001
Fasting glucose (mmol/L)	4.86 (0.42)	4.91 (0.60)	5.02 (0.46)	5.20 (0.87)	<0.001	<0.001
Total cholesterol (mmol/L)	4.68 (0.79)	4.63 (0.86)	4.82 (0.87)	4.91 (0.88)	<0.001	<0.001
Triglycerides (mmol/L)	0.79 (0.38)	0.94 (0.63)	1.01 (0.62)	1.26 (0.75)	<0.001	<0.001
LDL cholesterol (mmol/L)	2.75 (0.68)	2.78 (0.75)	2.99 (0.74)	3.14 (0.75)	<0.001	<0.001
HDL cholesterol (mmol/L)	1.55 (0.30)	1.45 (0.32)	1.41 (0.28)	1.32 (0.26)	<0.001	<0.001
TG/HDL-C	0.55 (0.36)	0.72 (0.70)	0.78 (0.64)	1.04 (0.81)	<0.001	<0.001
hsCRP (μg/mL)	0.37 (0.23)	3.48 (5.33)	0.51 (0.26)	3.10 (4.17)	<0.001	<0.001
FVC, %predicted	93.1 (12.2)	91.4 (12.5)	95.6 (12.7)	91.8 (12.3)	<0.001	0.836
FEV_1_, %predicted	95.8 (12.2)	93.1 (12.3)	96.7 (12.7)	93.1 (12.1)	<0.001	0.015
FEV_1_/FVC	89.2 (6.1)	88.4 (6.4)	87.4 (5.5)	87.7 (5.8)	<0.001	<0.001
Restrictive lung disease, n (%)^†^	330 (10.9)	84 (13.1)	47 (7.8)	75 (13.2)	0.008	0.664
**≥45** **y/o**						
Number	2014	468	465	358	—	—
Age, years	53.5 (6.8)	55.4 (7.0)	55.6 (7.1)	56.6 (7.3)	<0.001	<0.001
BMI (kg/m^2^)	22.5 (2.2)	23.4 (2.1)	27.4 (2.2)	28.3 (2.7)	<0.001	<0.001
Waist-to-height ratio	0.50 (0.05)	0.52 (0.04)	0.57 (0.04)	0.59 (0.05)	<0.001	<0.001
Body fat percentage, %	29.7 (4.5)	31.2 (3.9)	39.3 (2.9)	40.6 (3.6)	<0.001	<0.001
Mean arterial pressure (mmHg)	86.1 (12.9)	90.4 (12.4)	92.5 (13.0)	94.8 (13.1)	<0.001	<0.001
Fasting glucose (mmol/L)	5.24 (1.03)	5.62 (1.62)	5.49 (1.02)	5.96 (1.93)	<0.001	<0.001
Total cholesterol (mmol/L)	5.34 (0.98)	5.42 (1.11)	5.40 (1.04)	5.55 (0.98)	0.002	0.001
Triglycerides (mmol/L)	1.19 (0.74)	1.67 (2.59)	1.46 (1.19)	1.78 (1.23)	<0.001	<0.001
LDL cholesterol (mmol/L)	3.29 (0.82)	3.39 (0.87)	3.43 (0.86)	3.54 (0.84)	<0.001	<0.001
HDL cholesterol (mmol/L)	1.48 (0.31)	1.34 (0.30)	1.37 (0.27)	1.31 (0.29)	<0.001	<0.001
TG/HDL-C	0.89 (0.74)	1.48 (3.61)	1.17 (1.16)	1.50 (1.30)	<0.001	<0.001
hsCRP (μg/mL)	0.72 (0.48)	5.45 (6.78)	1.01 (0.50)	5.10 (5.21)	<0.001	<0.001
FVC, % predicted	93.9 (14.2)	90.4 (15.0)	91.6 (15.0)	86.5 (15.1)	<0.001	<0.001
FEV_1_, % predicted	96.0 (14.2)	92.9 (15.0)	94.3 (16.0)	89.5 (15.4)	<0.001	<0.001
FEV_1_/FVC	86.7 (6.2)	86.8 (6.3)	87.0 (5.6)	86.8 (5.1)	0.888	0.776
Restrictive lung disease, n (%)^†^	258 (12.8)	89 (19.0)	74 (15.9)	102 (28.5)	<0.001	<0.001

^†^Chi-squared test or Cochran–Armitage test for trend.

BMI, body mass index; TG, triglycerides; HDL-C, HDL cholesterol; FVC, forced vital capacity; FEV_1_, forced expiratory volume in 1 s. CRP, C-reactive protein.

Data are presented as mean (SD).

To evaluate the contribution of fat composition and distribution on the risk of RLD in women, multivariable logistic regression models were used to determine the effect of the aforementioned categorised groups (Table 4). In women aged <45 years, nonvisceral obesity decreased the risk of RLD (odds ratio [OR], 0.67; 95% confidence interval [CI], 0.56–0.80), even after adjustment for the confounding factors WHtR and MAP (OR, 0.77; 95% CI, 0.61–0.96), and it remained significant after further adjustment with FPG and CRP (OR, 0.76; 95% CI, 0.60–0.94). Although the visceral obesity group exhibited an increased risk of RLD, the association was not statistically significant. In the analysis for women aged ≥45 years, visceral obesity was significantly associated with an increased risk of RLD, with a crude OR of 2.83 (95% CI, 2.01–3.99). This increased effect remained significant after adjustment for the aforementioned confounding factors, WHtR/MAP and FPG/CRP, with ORs of 1.64 (95% CI, 1.06–2.54) and 1.60 (95% CI, 1.03–2.49), respectively.

**Table 4:** Joint effects of age and body fat distribution pattern on restrictive lung disease in women.

	Non-obese type	Non-visceral obesity type	Visceral obesity type
**<45** **y/o**			
No. of RLD/patient no. (%)	261/1952 (13.4%)	266/2843 (9.4%)	9/50 (18.0%)
OR and 95% CI			
Model 1^a^	1 (reference)	0.67 (0.56–0.80)*	1.42 (0.68–2.96)
Model 2^b^	1 (reference)	0.77 (0.61–0.96)*	2.17 (0.94–5.02)
Model 3^c^	1 (reference)	0.76 (0.60–0.94)*	1.99 (0.85–4.63)
**≥45** **y/o**			
No. of RLD/patient no. (%)	77/579 (13.3)	354/2422 (14.6)	92/304 (30.3)
OR and 95% CI			
Model 1^a^	1 (reference)	1.12 (0.86–1.45)	2.83 (2.01–3.99)*
Model 2^b^	1 (reference)	0.89 (0.67–1.19)	1.64 (1.06–2.54)*
Model 3^c^	1 (reference)	0.89 (0.67–1.19)	1.60 (1.03–2.49)*

Nonobese type: BF% ≤ 27.

Nonvisceral obesity type: BF% > 27 and visceral fat degree ≤10.

Visceral obesity type: BF% > 27 and visceral fat degree >10.

^a^Model 1, unadjusted analysis.

^b^Model 2, adjusted for waist-to-height ratio and mean arterial pressure.

^c^Model 3, Model 2+ fasting glucose level and hsCRP level.

RLD, restrictive lung disease, OR: odds ratio, CI: confidence interval, BF%: body fat percentage, CRP: C-reactive protein.

*Indicates *P* < 0.05.

## Discussion

This cross-sectional analysis examined the association of lung function with BF% and inflammation in a general population including 9,652 men and 8,150 women. This study demonstrated gender- and age-specific differences in BF% that could account for differences in inflammatory markers, at least CRP, and RLD prevalence between men and women. Lung function was significantly affected by body composition. The prevalence of RLD showed an increasing trend, whereas the values of FVC% and FEV_1_% significantly decreased along with the joint effect of BF% and inflammation in all men. Although age and the joint effect of BF% and CRP also contributed to lung function deterioration in women, the presence of RLD prevalence was complicated by CRP variations.

Cross-sectional population-based studies^25,26^ and a longitudinal study have reported^27^ that CRP is associated with lower lung function in men but not in women. Our findings further verify these results for men; however, our results were inconsistent for women. CRP showed effects on RLD prevalence in women and was more significant for those aged ≥45 years. The inconsistency may be partly explained by the diminished immunosuppressive effect of oestrogens on postmenopausal women because of differences in the ages of the populations in this study and other studies, which included younger subjects aged 28–56^25^, 20–29^28^, or 28 ± 6 years^27^.

CRP concentrations increase more rapidly as a function of greater accumulation of visceral adiposity and/or subcutaneous fat^29^. Moreover, we observed that the associations between the risk of RLD and nonvisceral obesity in women aged <45 years or between the risk of RLD and visceral adiposity in women aged ≥45 years were independent of CRP. After adjustment for confounding factors including CRP, visceral obesity significantly increased the risk of RLD in women aged ≥45 years. By contrast, nonvisceral obesity significantly reduced the risk of RLD for women aged <45 years.

Visceral fat has a high lipolytic rate that generates large amounts of free fatty acids that are delivered to the liver, causing increased hepatic glucose production, hyperinsulinemia, and metabolic syndrome (MetS)^30^. By contrast, accumulation of subcutaneous fat is independently associated with lower risk of mortality and disorders^31,32^. These findings are consistent with our findings that women with nonvisceral obesity and higher levels of subcutaneous fat are protected from lung function impairment that is associated with obesity; whereas men of all ages and women aged ≥45 years with higher amounts of visceral fat accumulation are at an increased risk of RLD.

Visceral adipose tissue deposition increases significantly with age in men and postmenopausal women. The decline in circulating oestrogens during menopause leads to a shift in adipose tissue deposition favouring the visceral depot. Studies have suggested that postmenopausal women can have up to twice the amount of visceral adipose tissue compared with premenopausal women^33^. In men, lung function according to FEV_1_/FVC ratio did not significantly vary by different percentiles of CRP/BF%. In addition, the prevalence of RLD was not affected by CRP but changed with BF%. Our findings may be explained by age and gender differences in the quantity and distribution of body fat that influence CRP differently between women and men.

Studies have demonstrated that lung function deteriorates with age in both men and women, but hormone therapy with exogenous oestrogens is associated with higher FEV_1_ and FVC in elderly women^34^. Hormone therapy with exogenous oestrogens can increase systemic inflammation^35^, suggesting that the female hormonal environment in women aged <45 years is somehow protective for lung function. In addition, obese men have lower androgen concentrations than do those with normal weight^36^. Although the mechanisms explaining the associations between sex hormones, body fat distribution, and inflammation require understanding, ample evidence exists supporting the differences in fatty acid mobilisation, oxidation, and storage between genders^37^ and the immunosuppressive effect of androgens^38^.

The observed results in this study are distinct but difficult to explain. Differences in hormones, adipose tissue, age, and other factors such as smoking are contributing factors associated with our findings. Systemic inflammation may be part of the pathophysiological process in the development of impaired lung function. Ageing is associated with a progressive increase in fat mass, in particular visceral adipose tissue, and a decrease in peripheral subcutaneous adipose tissue^39,40^. Gender differences in body fat distribution and systemic sex hormone concentrations play significant roles. Therefore, the mechanisms that lead to lung function disorders in obese individuals are still not completely clarified, and further studies are required.

Although our investigations demonstrated the associations between BF%/CRP and visceral obesity and the presence of RLD in a general population, the underlying physiological mechanisms are still unclear. In addition, the effects of CRP in studies are still controversial. Higher levels of CRP are associated with declined lung function FEV_1_ at baseline^41–44^, but the longitudinal association with lung function decline is not unanimous^42,44^. In another study, CRP exhibited a weak negative association with FEV_1,_ but it was no longer significant after correction for waist visceral fat. No association was observed between CRP and FVC in men with MetS^45^. Therefore, more studies are necessary to verify these observations.

Whether obesity has any effect on the structure of the airways, either through altered lipid deposition or remodelling, is still unknown. Although these questions may be best answered by direct studies of airway pathology, physiological investigations on the effects of weight loss would also be useful. Studies have showed that static lung volumes, but not exercise, improve with weight loss in middle-aged and older men, suggesting that altered lung function due to obesity is potentially modifiable^46^. A better understanding of the extent to which obesity modifies the physiological effects of lung function may improve our understanding of the relationship between lung function deterioration and obesity^10^.

Consistent with a previous study reporting that the coexistence of BF accumulation and low-grade inflammation is associated with impaired lung function for both genders in Korea^47^, our findings add to the evidence suggesting that body composition, systemic inflammation, fat distribution, and age are associated with the pathophysiological process in the development of impaired lung function and demonstrate the importance of sex-based research.

Our study had limitations. First, this was a cross-sectional study. Results from this study cannot prove a causality relationship between obesity, inflammatory markers, and lung function deterioration. Second, the results cannot be generalised to other ethnic populations or to obese groups because our results were obtained from a health check-up population of Chinese people. Third, only one CRP measurement per subject was tested, which may not reflect longitudinal inflammation status. Fourth, other proinflammatory mediators, such as leptin, that may be related to visceral fat were not measured. Fifth, although BIA is a cost-efficient and accessible method to measure body composition, it is limited in accurately measuring visceral fat deposition. Sixth, lifestyles that affect lung function such as smoking were not included in the analysis.

## Conclusions

In conclusion, the results of this study indicate significant gender differences in the joint effects of adiposity together with low-grade chronic inflammation, as BF% and CRP, on lung function in a sample of Chinese adults attending health check-ups. Variations of CRP concentrations contribute to lung function deterioration in different ways for men and women. This may be explained by changes in oestrogens and the endocrine functions of adipose tissue that produce hormones and cytokines, affect CRP levels, and consequently contribute to systemic inflammation. Visceral obesity increased the risk of RLD in women aged ≥45 years but not in those aged <45 years. Nonvisceral obesity reduces the risk of RLD in women aged <45 years. These observations suggest that visceral fat *per se* can be used as an effective parameter to assess the association between lung function and obesity.

### List of abbreviations

C-reactive protein, CRP; body fat percentage, BF%; chronic obstructive pulmonary disease, COPD; Body mass index, BMI; mean arterial pressure, MAP; fasting plasma glucose, FPG; total cholesterol, TC; triglycerides, TG; low-density lipopretein cholesterol, LDL-C; and high-density lipoprotein cholesterol, HDL-C; bioelectrical impedance analysis, BIA; forced vital capacity, FVC; forced expiratory volume in 1 second, FEV_1_; FEV_1_ to FVC ratio, FEV_1_/FVC; Restrictive lung disease, RLD; Waist-to-height ratio, WHtR; metabolic syndrome, MetS.

### Ethics approval and consent to participate

The study was approved by the Institutional Review Board of Chang-Gung Memorial Hospital and was conducted in accordance with the guidelines laid down in the Declaration of Helsinki.

## Data Availability

The datasets during and/or analysed during the current study available from the corresponding author on reasonable request.
